
JOURNAL INFORMATION
==============================
NLM Title Abbreviation: Atlanta Med Surg J (1884)
NLM Journal ID: 101251623

Atlanta Medical and Surgical Journal (1884)

Publisher: Biomedical Journal Digitization Project

ARTICLE INFORMATION
==============================
PMCID: PMC9201837
PMID: 35829597
Article version: 1
Subjects: Original Communications

The Treatment of Fractures

Print publication date: 1899 Jan
Volume: 15
Volume Series: New Ser
Issue: 11
First page: 742
Last page: 742
License: This work is free of known copyright restrictions. For more information, please see PMC Back Issue Digitization.
License URL: https://creativecommons.org/publicdomain/mark/1.0/

==============================

*To fill vacancy in the Council*—Dr. L. McLane Tiffany, Baltimore, Md.

Place of meeting, New Orleans; time, to be determined by the Secretary with advice of the Council.

After the presentation and adoption of resolutions of thanks, the Association adjourned.

---

### THE TREATMENT OF FRACTURES.

Dr. W. L. Estes (*International Journal of Surgery*) says:

First. Unless a fragment is threatening to break through the skin it should never be reduced except by the physician, and then only when the parts can be kept in permanent apposition.

Second. Men carrying an injured person should not keep step, as the jar is greater.

Third. Strychnia should be given for shock, morphia for pain, but no alcohol.

It is very rarely necessary to make a patient go through the double agony of temporary and permanent setting of the broken bones.

In simple fracture gentle rubbing of the ends will assist in getting rid of shreds of tissue which are invariably caught there.

Nowadays a surgeon will rarely be satisfied that a bone is properly set until verified by the X-rays.

Plastic splints, preferably plaster of Paris, are the best apparatus.

No simple fractures require constant confinement to bed, except of the innominata and upper third of the femur.

Do not wait for the swelling to disappear before putting on a permanent dressing.

A well-applied splint with good apposition of the fragments should not be removed too early. Proper time for massage is two or three weeks after fracture of upper extremities and four or five weeks for lower extremities.

---

**NOTICE.—This JOURNAL and THE INTERNATIONAL JOURNAL OF SURGERY one year for $2.00 cash.**
